# The role of group II metabotropic glutamate receptors in cognition and anxiety: Comparative studies in *GRM2*^*−/−*^, *GRM3*^*−/−*^ and *GRM2/3*^*−/−*^ knockout mice

**DOI:** 10.1016/j.neuropharm.2014.08.010

**Published:** 2015-02

**Authors:** Bianca De Filippis, Louisa Lyon, Amy Taylor, Tracy Lane, Philip W.J. Burnet, Paul J. Harrison, David M. Bannerman

**Affiliations:** a Section of Behavioural Neuroscience, Department of Cell Biology and Neuroscience, Istituto Superiore di Sanita, Viale Regina Elena, 299, I-00161 Rome, Italy; b Department of Psychiatry, University of Oxford, Warneford Hospital, Oxford, UK; c Department of Experimental Psychology, University of Oxford, Oxford OX1 3UD, UK

**Keywords:** Hippocampus, Spatial memory, Anxiety, Arousal

## Abstract

Group II metabotropic glutamate receptors (mGlu2 and mGlu3, encoded by *GRM2* and *GRM3*) have been implicated in both cognitive and emotional processes, although their precise role remains to be established. Studies with knockout (KO) mice provide an important approach for investigating the role of specific receptor genes in behaviour. In the present series of experiments we extended our prior characterisation of *GRM2/3*^*−/−*^ double KO mice and, in complementary experiments, investigated the behavioural phenotype of single *GRM2*^*−/−*^ and *GRM3*^*−/−*^ mice. We found no consistent effect on anxiety in either the double or single KO mice. The lack of an anxiety phenotype in any of the lines contrasts with the clear anxiolytic effects of mGlu2/3 ligands. Motor co-ordination was impaired in *GRM2/3*^*−/−*^ mice, but spared in single *GRM2*^*−/−*^ and *GRM3*^*−/−*^ mice. Spatial working memory (rewarded alternation) testing on the elevated T-maze revealed a deficit in *GRM2*^*−/−*^ mice throughout testing, whereas *GRM3*^*−/−*^ mice exhibited a biphasic effect (initially impaired, but performing better than controls by the end of training). A biphasic effect on activity levels was seen for the *GRM2*^*−/−*^ mice. Overall, the phenotype in both *GRM2*^*−/−*^ and *GRM3*^*−/−*^ mice was less pronounced – if present at all – compared to *GRM2/3*^*−/−*^ mice, across the range of task domains. This is consistent with possible redundancy of function and/or compensation in the single KO lines. Results are discussed with reference to a possible role for group II metabotropic glutamate receptors at the interface between arousal and behavioural performance, according to an inverted U-shaped function.

## Highlights

• *GRM2*^*−/−*^ mice exhibited impaired spatial short-term memory (rewarded alternation).
• *GRM3*^*−/−*^ mice displayed bi-directional effects on this spatial short-term memory task.
• *GRM2*^*−/−*^ mice displayed bi-directional effects on activity levels.
• There was no consistent anxiety effect in either double or single knockout mice.
• Behavioural phenotypes were weaker (or absent) in single than in double knockout mice.

## 1 Introduction

Group II metabotropic glutamate receptors (mGlu2 encoded by *GRM2* and mGlu3 encoded by *GRM3*) are G-protein coupled receptors that inhibit adenylate cyclase (Niswender and Conn, 2010). A key function of group II mGlu receptors is to act pre-synaptically to inhibit neurotransmitter release, and mGlu2 receptors act primarily as autoreceptors to modulate the release of glutamate (Anwyl, 1999, Cartmell and Schoepp, 2000, Schoepp, 2001), whilst mGlu3 are also glial. Group II mGlu receptors have been implicated in both cognitive and emotional processes, and have been linked with a number of neuropsychiatric conditions, including anxiety, stress-related disorders, schizophrenia and substance misuse (Harrison et al., 2008, Markou, 2007, Moghaddam, 2004, Swanson et al., 2005). Nevertheless, the precise role that these receptors play in cognition and emotion remains to be fully established.

Genetically modified mice represent an important tool for investigating the role of different receptor subtypes in behaviour (Bannerman, 2009). In order to elucidate the role of group II mGlu receptors in cognition we have studied *GRM2/3*^*−/−*^ double KO mice, lacking both mGlu2 and mGlu3 (Lyon et al., 2011). By studying these double KO mice we aimed to circumvent any possible compensatory changes and/or redundancy of function between these two homologous receptor subtypes (Lyon et al., 2008).

We previously reported that *GRM2/3*^*−/−*^ mice exhibit a distinct pattern of cognitive impairments across a range of hippocampus-dependent spatial memory tests (Lyon et al., 2011). *GRM2/3*^*−/−*^ mice were impaired on appetitively motivated spatial memory tests (e.g. spatial working and reference memory on the radial maze), and on tests which rely on spontaneous exploratory behaviours (e.g. spatial novelty preference in a Y-maze), but in contrast they performed as well as wild-type (WT) controls on aversively motivated spatial memory paradigms like the Morris water maze. Indeed, *GRM2/3*^*−/−*^ mice were impaired on an appetitive version of a Y-maze spatial reference memory task but normal on a swim-escape version of the same task. *GRM2/3*^*−/−*^ mice were hypoactive when tested in photocell activity cages, leading us to suggest reduced levels of arousal. Furthermore, while injection stress impaired spatial working memory performance on an appetitive task in WTs, it actually improved the performance of the *GRM2/3*^*−/−*^ mice, consistent with an altered arousal–cognition relationship in these animals. An important question that remains concerns the relative contributions of mGlu2 and mGlu3 to these processes.

A number of lines of evidence, mainly from pharmacological studies, have suggested a role for group II mGlu receptors in anxiety (Harvey and Shahid, 2012, Swanson et al., 2005). An anxiolytic effect of mGlu2/3 agonists has been demonstrated in a number of rodent anxiety paradigms [e.g. elevated plus maze (Linden et al., 2005); fear potentiated startle (Helton et al., 1998); lactate-induced panic (Shekhar and Keim, 2000)], and confirmed in healthy human volunteers in the fear potentiated startle test (Grillon et al., 2003), in panic disorder patients exposed to CO_2_-induced anxiety (Schoepp et al., 2003) and in patients with general anxiety disorder (Dunayevich et al., 2008). Furthermore, mGlu2 selective positive allosteric modulators are now being developed which also display anxiolytic/antidepressant properties (Fell et al., 2011). Interestingly (although somewhat counter-intuitively), mGlu2/3 antagonists have also been suggested for the treatment of anxiety (Iijima et al., 2007, Shimazaki et al., 2004, Yoshimizu et al., 2006), although results are more equivocal. Against this, studies in genetically modified mice lacking either mGlu2 (*GRM2*^*−/−*^ mice) or mGlu3 (*GRM3*^*−/−*^ mice) have reported no anxiety phenotype (Fell et al., 2011, Linden et al., 2005, Morishima et al., 2005). One possible explanation for these null results is compensatory changes in gene expression and/or redundancy of function between these two homologous receptor subtypes for the anxiety phenotype in the single KO lines (Lyon et al., 2008), whereas pharmacological ligands will activate or inhibit both mGlu2 and mGlu3.

Therefore, in the present study we assessed anxiety in *GRM2/3*^*−/−*^ mice (thus circumventing the possibility of compensatory changes/redundancy of function), using ethologically based, unconditioned tests, as part of a more extensive test battery investigating sensorimotor, motivational and emotional behaviours. This builds upon and extends our previous work characterising hippocampus-dependent cognitive behaviours in these mice (Lyon et al., 2011). For comparison, we also evaluated each of the single KOs (*GRM2*^*−/−*^ and *GRM3*^*−/−*^ mice) against their respective WT littermate controls, using the same battery of tests. In addition, we assessed the *GRM2*^*−/−*^ and *GRM3*^*−/−*^ mice on key spatial memory tests employed in our previous studies with *GRM2/3*^*−/−*^ mice (Lyon et al., 2011). Thus, spatial working/short-term memory was assessed in *GRM2*^*−/−*^ and *GRM3*^*−/−*^ mice using both appetitively motivated, rewarded alternation on the T-maze and a spontaneous, exploratory driven spatial novelty preference task in the Y-maze. Long-term spatial memory was assessed using both appetitive and aversive/swim-escape versions of the Y-maze reference memory task.

## 2 Materials and methods

### 2.1 Subjects

Adult male mice (>2.5 months) on a C57BL/6J background were obtained from GlaxoSmithKline, Harlow, UK. Single *GRM2*^*−/−*^ and *GRM3*^*−/−*^ mice, and their respective WT littermate controls, were generated as previously described (Corti et al., 2007, Yokoi et al., 1996). Separate lines of WT and *GRM2/3*^*−/−*^ mice were produced as in Lyon et al. (2011). Group sizes varied between experiments and lines, and are given in the relevant Tables and Figures. Mice were 2.5–4 months old at the start of the anxiety/sensorimotor test battery, and 4–5.5 months old at the end of this test battery. They then began spatial memory testing, and were 7–8 months old at the end of the spatial memory tests.

Animals were housed in groups of 2–4 and kept on a 12-h light–dark cycle (lights on at 07:00 and off at 19:00), with all testing conducted during the light phase. For the battery of anxiety and motor tests, and for the aversively motivated tasks, mice were given *ad libitum* access to food and water. For all appetitively motivated tasks, mice were maintained on a restricted feeding schedule at not less than 90% of their free-feeding weight. For several days prior to the start of each appetitive test, mice were habituated to the maze and to drinking the sweetened condensed milk (diluted 50:50 with water) that was used as a reward. Habituation was conducted in a room other than the experimental test room. Behavioural experiments were conducted under the auspices of U.K. Home Office Project and Personal licenses held by the authors, and the study was approved by the local ethics committee.

### 2.2 Genotyping

Genotypes of all animals were confirmed by the “Mouse genotyping group” at GSK, Harlow. *GRM2* fragments were amplified using forward primer (CTG TCT CTC TAT CTC TCT GC) and reverse primer (TGT GTG TGT GTA ACA TGA TGG). PCRs were performed with a denaturing step at 95 °C (15 min) then 94 °C (30 s), followed by annealing at 60 °C (90 s) and extension at 72 °C (1 min). After 35 cycles, the reaction was maintained at 72 °C for a further 10 min, and the products resolved on a 2% agarose gel. The WT product was a single 900 bp band, and the KO product a 450 bp band. *GRM3* genotyping yielded a 2 kbp product in WT and a 500 bp product in KO. The disparity in size prevented the two fragments from being amplified in a single multiplex PCR. Two separate PCRs were therefore conducted, one for the WT product (forward primer: GTT TCT AGG ACT TCC TAT GG; reverse primer: AAC GAT GCT CTG ACA AAC TCC) and a second for the KO product (forward primer: CGT ACG TCG GTT GCT ATG G; reverse primer: GTC AGA TAT AGT GAG AGC AGG). Both PCRs were performed with a denaturing step at 95 °C (15 min) then 94 °C (30 s), followed by annealing at 56 °C (90 s) and extension at 72 °C (150 s). After 35 cycles, the reaction was maintained at 72 °C for 10 min. The absence of GRM2 mRNA in founding members of the *GRM2*^*−/−*^ population was confirmed by the use of *in situ* hybridisation histochemistry (Yokoi et al., 1996), as was the absence of GRM3 mRNA in the founding members of the *GRM3*^*−/−*^ population (Corti et al., 2007).

### 2.3 Order of testing

All mice were experimentally naïve at the start of the test battery. The order of testing was as follows: Black & White Alley; Neophagia (first version); Open field; Horizontal bar; rotarod test; Multiple Static Rods; Elevated Plus Maze, Neophagia (second version); Spatial novelty preference test; Locomotor activity; Appetitive spatial working memory T-maze, Appetitive/aversive spatial reference memory (SRM) Y-maze (with test order counterbalanced for the two versions of the reference memory Y-maze task). There was a minimum of at least 24 h between each test. Comparisons of *GRM2/3*^*−/−*^, *GRM2*^*−/−*^ and *GRM3*^*−/−*^ mice against their respective WT control groups were conducted separately, and by different experimenters. All behavioural testing took place during the light phase of the day between 9.00 am and 6.00 pm. Data from the *GRM2/3*^*−/−*^ mice on spontaneous locomotor activity and the spatial memory tests has been published previously (Lyon et al., 2011).

### 2.4 Emotionality tests

Anxiety was assessed using a number of ethological, unconditioned tests, which assessed approach/avoidance conflict in these mice.

#### 2.4.1 Black & White Alley

The Black & White Alley (120 cm × 9 cm, 29 cm high walls) was painted black from one end to the middle, and white from the other end to the middle. The mouse was placed into the alley at the end of the black arm and observed for two minutes. Variables measured were latency to enter the white arm (which is regarded as more anxiogenic than the black arm), total amount of time spent in the white arm, and number of crossings between the two arms.

#### 2.4.2 Neophagia

This test exploits the natural wariness of rodents to consume novel foodstuffs. To overcome potential floor/ceiling effects, two versions of the task were performed with increasing anxiogenic character. In each, the mouse was placed in a novel environment (underneath an upturned plastic jug/in a closed arm on an elevated Y-maze) and presented with a completely novel food (sweetened condensed milk/Noyes reward pellets). All mice were food deprived the night before for approximately 15 h. Variables measured were latency to contact, and then to consume, the novel food. A maximum of 3 min was allowed for each trial.

#### 2.4.3 Elevated plus maze

The elevated plus maze consisted of four 27 cm long, 8 cm wide alleys connected by a central platform to form a plus-shape. Two of the alleys had high (30 cm) walls, and two had low (0.5 cm) walls. The high walled alleys (“closed arms”) are presumed to be less anxiogenic than the low walled ones (“open arms”), while the central platform (“junction”) (12.5 cm by 6 cm) remains relatively neutral. The apparatus was placed 70 cm above the ground. The mouse was placed on the central platform and electronically tracked for five minutes. Variables measured were latency to enter a closed arm, latency to enter an open arm, percentage of time spent in each area of the maze (open vs closed vs junction), and total distance covered within the maze.

#### 2.4.4 Anxiogenic open field

The open field comprised a brightly illuminated circular arena (a white metallic drum), with diameter 60 cm. The “centre” of the open field was defined as a circular area with an outer edge 19 cm from the edge of the arena. The mouse was placed inside the arena facing the sidewall and electronically tracked for five minutes. Variables measured were percentage of time in the central area versus the outer area, and total distance covered in the arena.

### 2.5 Motor tests

#### 2.5.1 Horizontal bar

The horizontal bar was 0.2 cm in diameter, 38 cm long, and made of metal. It was attached to two wooden supports, and positioned 49 cm above the padded bench surface. The mouse was placed with its front paws touching the bar and then quickly released, so that it grasped the bar at its central point. Performance was scored based on the length of time that the mouse held onto the bar: a score of 5 was given for holding on for 30 s or for traversing the bar to reach one of the wooden supports; 4 for holding on for 21–30 s; 3 for 11–20 s; 2 for 6–10 s; and 1 for 0–5 s.

#### 2.5.2 Accelerating rotarod

The rotarod was a 3.5 cm wide (diameter), 4.5 cm long knurled rod, attached at each end to 30 cm diameter flanges. The rod was positioned 17 cm above the padded bench surface. Speed and acceleration of the rotarod were electronically controlled. The mouse was held by the tail and allowed to grasp the rod. Initially the rod rotated at 4 rpm. If the mouse was still in place after ten seconds, the rotation of the rod was gradually accelerated at a rate of 20 rpm^2^. The latency to fall from the rod and the speed of rotation at which the mouse fell were recorded.

#### 2.5.3 Multiple static rods

The multiple static rods consisted of five 60 cm long wooden rods attached perpendicularly to a supporting beam. The rods were arranged in order of decreasing diameter: rod 1 was 3.3 cm wide; rod 2, 2.7 cm; rod 3, 2.1 cm; rod 4, 1.4 cm; rod 5, 0.8 cm. The rods were elevated 60 cm above a padded surface. On each trial the mouse was placed 2 cm from the distal end of the rod, facing away from the supporting beam, and the time taken for the mouse to turn 180° to face the supporting beam, recorded. These “orientation times” are sensitive to small deficits in motor co-ordination. The time taken for the mouse to run 60 cm along the beam to reach the refuge of the support (“transit time”) was also recorded. A maximum of 5 min was allowed for each rod. The mouse was placed on the largest diameter rod first, continuing with the next largest on each subsequent trial.

### 2.6 Spontaneous locomotor activity

Spontaneous locomotor activity was measured during a two-hour period in the light phase (12 pm–2 pm). All mice were placed singly into a transparent plastic cage (26 cm × 16 cm × 17 cm) with a ventilated lid. Two infrared photocell beams crossed the cage 1.5 cm above the floor, with each beam 7 cm from the centre of the cage. Mice were left in a quiet room with the lights on for 2 h. The number of beam breaks made by each mouse was recorded in 24 bins of 5 min.

### 2.7 Cognitive tests

#### 2.7.1 Spontaneous spatial novelty preference task

Single KO and WT mice were compared on a spontaneous, spatial novelty preference task in which behaviour is driven, not by an overt unconditioned stimulus (US; e.g. a food reward), but instead relies upon animals' natural exploratory drive. This task therefore provides a non-aversive experimental context but performance does not rely on the motivating or rewarding effects of food. We previously showed that *GRM2/3*^*−/−*^ double KO mice exhibit a reduced spatial novelty preference on this task (Lyon et al., 2011), and we repeated the test in *GRM2/3*^*−/−*^ mice here.

The Y-maze was made from transparent Perspex, and consisted of three 30 cm long, 8 cm wide arms with 20 cm high walls, connected by a central junction. A thin layer of sawdust covered the floor of the maze. Each mouse was assigned two arms (the “start arm” and the “other arm”) to which they were exposed during the first phase of the task (the “exposure phase”). Allocation of arms to specific spatial locations was counterbalanced within each genotype. During the 5-min “exposure” phase, the entrance to the third, “novel”, arm was closed off by the presence of a large Perspex block. The mouse was placed at the end of the start arm, facing the experimenter, and allowed to explore the start arm and the other arm freely for five minutes, beginning as soon as the mouse left the start arm. The number of entries into each arm and the length of time spent there were recorded. At the end of the five minutes, the mouse was removed from the maze and returned to the home cage for one minute. During this time, the Perspex block closing off the novel arm was removed and the sawdust redistributed throughout the maze to minimise the use of odour cues. The mouse was then returned immediately to the start arm, facing the experimenter, for the 2-min test phase. This consisted of two minutes free exploration during which the mouse could enter all 3 arms, beginning as soon as the mouse left the start arm.

The amount of time that the mouse spent in each arm, and the number of entries into each arm, were recorded, during both the exposure and the test phase. For the test phase, a discrimination ratio [(novel arm)/(novel + other arm)] was calculated both for number of arm entries and time spent in each arm. Previous work in this laboratory has demonstrated that WT mice display a marked preference for the novel arm during the test phase, and that this preference relies on extramaze cues. This preference for the novel arm is abolished in mice with cytotoxic hippocampal lesions (Sanderson et al., 2007).

#### 2.7.2 Spatial working memory on the elevated T-maze

Our previous study demonstrated that spatial working memory on the elevated T-maze is impaired in *GRM2/3*^*−/−*^ mice (Lyon et al., 2011). The T-maze consisted of a wooden start arm (47 × 10 cm) and two identical goal arms (35 × 10 cm), surrounded by a 10 cm high wall. A food well was positioned 3 cm from the end of each goal arm, and the whole maze was surrounded by prominent distal extramaze cues. Mice received five trials per day for six days, with an ITI of approximately forty minutes. For analysis and presentation, data are presented as 3 blocks of 10 trials, having combined data from consecutive days. Each trial consisted of a sample run followed by a choice run. On the sample run, mice were forced either left or right (chosen pseudorandomly with equal numbers of left and right turns, and no more than three consecutive turns in any direction) by the presence of a large wooden block, closing off one of the goal arms. At the end of the goal arm the mouse collected a reward of 0.1 ml sweetened condensed milk. The block was then removed and the mouse placed back in the start arm, facing the experimenter, for the choice run. The mouse could now select either goal arm but was rewarded only for choosing the arm that had not been visited on the sample run, i.e., it was rewarded for alternating (non-matching to place). The interval between the sample run and the choice run was approximately 5 s. The number of correct alternations was recorded for each mouse. In addition, we recorded latencies for both the sample runs and the choice runs. We recorded the latency of the mice to run (i) from the beginning of the start arm to the food well on the sample trial, and (ii) from the beginning of the start arm until making a choice into one of the goal arms on the choice trial. Spatial working memory performance on the T-maze is dependent on the hippocampus (Deacon et al., 2002).

#### 2.7.3 Comparison of appetitively and aversively motivated spatial reference memory on the Y-maze

The aim of the experiment was to compare performance of WT and single KO mice on appetitively and aversively motivated versions of the Y-maze spatial reference memory task, with the order of testing and the experimental rooms in which the tests were performed (and therefore the spatial cues available), fully counterbalanced in order to control for practice effects or differences in the salience of the available spatial cues (see also Lyon et al., 2011). Thus, 50% of WT and 50% of KO mice first performed the appetitive Y-maze SRM task in room A. These animals then performed an aversively motivated Y-maze SRM task in room B. Conversely, the remaining 50% began with the aversively motivated Y-maze task in room A, followed by the appetitively motivated Y-maze task in room B. We have previously shown that the *GRM2/3*^*−/−*^ double KO mice are impaired on the appetitive, but not the aversive, version of the task (Lyon et al., 2011).

#### 2.7.4 Appetitively motivated spatial reference memory on the elevated Y-maze

The elevated Y-maze consisted of three identical wooden arms, each 50 cm long by 9 cm wide, with a low wall (0.5 cm), connected by a central polygonal platform (14 cm diameter). A food well was positioned at the end of each arm. Each mouse was assigned a goal arm, defined by its position relative to extramaze spatial cues, which was baited with 0.1 ml sweetened condensed milk on all trials. On each trial, the mouse was placed at the end of one of the two non-baited arms (the “start arm”), facing the experimenter; 50% of trials began from the arm to the right of the goal arm, and 50% from the arm to the left. Neither arm was used as the start arm for more than three consecutive trials. Allocation of start and goal arms was counterbalanced across groups. Having been placed at the end of the start arm, the mouse was allowed to choose one of the remaining arms. If it chose the goal arm, it was allowed to consume the milk reward before being returned to the home cage. Mice that chose incorrectly were returned to the home cage immediately. To prevent the use of intramaze cues, the entire maze was rotated periodically (approximately every 5 trials). Mice received ten trials per day for 6 days, with an inter-trial interval (ITI) of approximately five minutes. The last block of ten trials was conducted using post-choice reinforcement: the condensed milk reward was added to the food well only after the mouse had made a choice, to ensure that the animals were not locating the milk by virtue of its odour. Previous work in this laboratory using the same maze has demonstrated that this task is hippocampus-dependent in mice (Deacon et al., 2002).

#### 2.7.5 Aversively motivated spatial reference memory in the Y-maze

The Y-maze was made from transparent Perspex, and consisted of three 30 cm long, 8 cm wide arms with 20 cm high walls, connected by a central junction. The maze was filled with water (temperature 21 °C ± 1 °C) to a depth of approximately 12 cm which obliged the mice to swim. Mice could escape from the water by climbing onto a platform (8 cm by 8 cm) hidden approximately 1.5 cm below the water surface in one of the arms of the maze. Milk was added to the water to prevent the mice from seeing the platform. Mice received five trials per day in this deep water escape Y-maze task for six days. On each trial the mouse was allowed 90 s to find the platform; any that failed to do so were guided there by the experimenter. Mice were allowed to rest on the platform for 30 s before being transferred to a heated cage. On day seven (24 h after training trial 30), a transfer test was performed, analogous to that used in the water maze, in order to assess the extent of any spatial memory for the platform location. The platform was removed from the maze and the mouse allowed to swim freely for 30 s. Time spent searching in each arm was recorded. Previous work in this laboratory has confirmed that the “swimming Y-maze” task, like the appetitive Y-maze, is hippocampus-dependent (unpublished).

### 2.8 Statistical analysis

When comparing the performance of two groups of mice, parametric data were analysed using *t*-tests, or with a repeated measures ANOVA if there was a within-subjects factor. Where data violated the assumptions of normality or equality of variance, which are required for parametric analysis (i.e. the data were non-parametric), then Mann–Whitney *U*-tests were performed for simple group comparisons with no within-subjects factors. For non-parametric data sets which included both between and within-subjects factors (e.g. multiple static rods data collected across different sized rods), the data were first transformed using a log_10_ transformation to satisfy the assumptions of normality and equality of variance, and then analysed using a two way, repeated measures ANOVA.

## 3 Results

All animals displayed normal appearance and no gross abnormalities in home-cage behaviours (assessed during short, non-systematic observations by the experimenter during the light period). Body weight of *GRM2/3*^*−/−*^ and *GRM3*^*−/−*^ mice did not differ from their respective WT controls (*GRM2/3*^*−/−*^, *n* = 20, 28.0 g ± 0.5 g vs. WT, *n* = 19, 28.5 g ± 0.3 g; *t* < 1; *p* > 0.20; *GRM3*^*−/−*^, *n* = 15, 26.7 g ± 0.3 g vs. WT, *n* = 15, 27.1 g ± 0.5 g; *t* < 1; *p* > 0.50). *GRM2*^*−/−*^ mice were slightly, but significantly, lighter than their WT littermates at the start of the test battery (*GRM2*^*−/−*^, *n* = 14, 27.1 g ± 0.4 g, vs. WT, *n* = 15, 28.8 g ± 0.6 g; *t*(27) = 2.23; *p* < 0.05). However, this difference was short-lasting and no longer evident at the time of rotarod testing.

### 3.1 Measures of emotionality

The results of the emotionality tests are summarised in Table 1, Table 2, Table 3, Table 4, Table 5, Table 6. Overall the tests revealed no major differences in emotionality between *GRM2/3*^*−/−*^ and WT mice (Table 1, Table 4). Similarly, no consistent differences in anxiety-like behaviours were evident between either *GRM2*^*−/−*^ (Table 2, Table 5) or *GRM3*^*−/−*^ mice (Table 3, Table 6), and their respective controls.

**Table 1:** Comparison of *GRM2/3*^*−/−*^ and wild-type (WT) mice in the Black & White Alley, the Elevated Plus Maze and Open Field.

Task & measure	WT	*GRM2/3*^*−/−*^	Statistics
**Black & White Alley**			
Total time in white arm (s)	45.9 ± 1.6	42.4 ± 2.1	*t*(25) = 1.34; *p* = 0.191
Number of crossings	9.5 ± 0.8	8.6 ± 0.8	*t* < 1; *p* > 0.20
**Elevated Plus Maze**			
% time in open arms	26.4 ± 1.6	23.1 ± 2.3	*t*(30) = 1.2; *p* = 0.239
Latency to enter open arms (s)	9.6 ± 2.8	11.5 ± 2.6	*t* < 1; *p* > 0.20
Total distance travelled (cm)	2229.1 ± 60.2	2322.9 ± 89.4	*t* < 1; *p* > 0.20
Number of open arm entries	10.5 ± 0.5	11.3 ± 0.9	*t* < 1; *p* > 0.20
**Open Field**			
Time in centre (s)	17.1 ± 2.0	15.3 ± 1.9	*t* < 1; *p* > 0.20
Latency to enter centre (s)	66.1 ± 10.5	68.7 ± 8.4	*t* < 1; *p* > 0.20
Total distance travelled (cm)	4106.5 ± 102.6	3880.0 ± 204.8	*t*(30) = 1.06; *p* = 0.299

Data are presented as mean ± SEM and were analysed with *t*-tests. For the Black & White Alley, *n* = 16 WT; *n* = 11 *GRM2/3*^*−/−*^; for the other tests, *n* = 18 WT; *n* = 14 *GRM2/3*^*−/−*^.

**Table 2:** Comparison of *GRM2*^*−/−*^ and wild-type (WT) mice in the Black & White Alley, the Elevated Plus Maze and Open Field.

Task & measure	WT	*GRM2*^*−/−*^	Statistics
**Black & White Alley**			
Total time in white arm (s)	40.2 ± 2.6	37.5 ± 2.8	*t* < 1; *p* > 0.40
Number of crossings	8.7 ± 0.9	8.6 ± 0.8	*t* < 1; *p* > 0.90
**Elevated Plus Maze**			
% time in open arms	11.1 ± 1.9	12.5 ± 1.9	*t* < 1; *p* > 0.50
Latency to enter open arms (s)	25.1 (10.6–37.6)	11.5 (1.7–20.6)	*U* = 72; *p* = 0.150
Total distance travelled (cm)	1410.8 ± 116.4	1543.2 ± 101.0	*t* < 1; *p* > 0.40
Number of open arm entries	9.6 ± 1.3	11.1 ± 1.3	*t* < 1; *p* > 0.40
**Open Field**			
Time in centre (s)	7.3 ± 0.8	5.6 ± 0.5	*t*(27) = 1.76; *p* = 0.089
Latency to enter centre (s)	28 (24.6–38.5)	20.2 (6.53–57.4)	*U* = 78; *p* = 0.239
Total distance travelled (cm)	3314.3 ± 155.1	3119.2 ± 142.9	*t* < 1; *p* > 0.30

Parametric data are presented as mean ± SEM and were analysed with *t*-tests. Non-parametric data are presented as median (inter-quartile range) and were analysed with Mann–Whitney *U*-tests. *n* = 15 WT; *n* = 14 *GRM2*^*−/−*^.

**Table 3:** Comparison of *GRM3*^*−/−*^ and wild-type (WT) mice in the Black & White Alley, the Elevated Plus Maze and Open Field.

Task & measure	WT	*GRM3*^*−/−*^	Statistics
**Black & White Alley**			
Total time in white arm (s)	39.6 ± 1.9	34.7 ± 2.1	*t*(28) = 1.68; *p* = 0.104
Number of crossings	8.7 ± 0.7	9.7 ± 0.7	*t*(28) = 1.03; *p* = 0.312
**Elevated Plus Maze**			
% time in open arms	7.4 ± 0.8	9.5 ± 0.9	*t*(28) = 1.71; *p* = 0.099
Latency to enter open arms (s)	18.1 (5.4–55.8)	3.0 (1.6–4.6)	*U* = 58; *p* = 0.024*
Total distance travelled (cm)	2332.2 ± 71.6	2202.5 ± 83.3	*t*(28) = 1.18; *p* = 0.248
Number of open arm entries	17.9 ± 2.4	25.2 ± 3.1	*t*(28) = 1.88; *p* = 0.070
**Open Field**			
Time in centre (s)	6.2 ± 0.6	6.5 ± 0.8	*t* < 1; *p* > 0.70
Latency to enter centre (s)	50.7 ± 12.1	25.3 ± 5.7	*t*(28) = 1.89; *p* = 0.068
Total distance travelled (cm)	3041.3 ± 125.3	3305.3 ± 163.5	*t*(28) = 1.28; *p* = 0.211

*p < 0.05. Parametric data are presented as mean ± SEM and were analysed with *t*-tests. Non-parametric data are presented as median (inter-quartile range) and were analysed with Mann–Whitney *U*-tests. *n* = 15 WT; *n* = 15 *GRM3*^*−/−*^.

**Table 4:** Comparison of *GRM2/3*^*−/−*^ and wild-type (WT) mice in two versions of the Neophagia Test.

Task & measure	WT	*GRM2/3*^*−/−*^	Statistics
**Neophagia I (Milk/Jug)**			
Latency to contact (s)	4 (3–6)	4 (3–5)	*U* = 209.5; *p* = 0.518
Latency to drink (s)	13 (9.8–17)	13 (9–16)	*U* = 198; *p* = 0.834
Latency to drink – latency to contact (s)	7 (4–9.5)	8 (5–11.5)	*U* = 173.5; *p* = 0.494
**Neophagia II (Noyes/Y-maze)**			
Latency to contact (s)	10.3 ± 1.2	9.2 ± 1.2	*t* < 1; *p* > 0.20
Latency to eat (s)	109.1 ± 23.3	51.8 ± 11.0	*t*(30) = 2.03; *p* = 0.051
Latency to eat – latency to contact (s)	61(14.5–139.8)	21 (12.3–50.8)	*U* = 227.5; *p* = 0.184

Parametric data are presented as mean ± SEM and were analysed with *t*-tests. Non-parametric data are presented as median (inter-quartile range) and were analysed with Mann–Whitney *U*-tests. *n* = 14 WT; *n* = 11 *GRM2/3*^*−/−*^ for test 1; *n* = 18 WT; *n* = 14 *GRM2/3*^*−/−*^ for tests 2.

**Table 5:** Comparison of *GRM2*^*−/−*^ and wild-type (WT) mice in two versions of the Neophagia Test.

Task & measure	WT	*GRM2^−/−^*	Statistics
**Neophagia I (Milk/Jug)**			
Latency to contact (s)	1 (1–4)	1 (1–1)	*U* = 73; *p* = 0.162
Latency to drink (s)	27 (8–93)	11 (3–45.5)	*U* = 87; *p* = 0.432
Latency to drink – latency to contact (s)	26 (6–77)	10 (1.25–43)	*U* = 74.5; *p* = 0.183
**Neophagia II (Noyes/Y-maze)**			
Latency to contact (s)	28 (17.5–32)	30.5 (18.5–44.75)	*U* = 84.5; *p* = 0.371
Latency to eat (s)	313 (199–360)	274 (139–359.75)	*U* = 85; *p* = 0.383
Latency to eat – latency to contact (s)	200 (176–330)	223 (104.25–314.5)	*U* = 94; *p* = 0.631

Data are presented as median (inter-quartile range) and were analysed with Mann–Whitney *U*-tests. *n* = 15 WT; *n* = 14 *GRM2*^*−/−*^.

**Table 6:** Comparison of *GRM3*^*−/−*^ and wild-type (WT) mice in two versions of the Neophagia Test.

Task & measure	WT	*GRM3^−/−^*	Statistics
**Neophagia I (Milk/Jug)**			
Latency to contact (s)	3 (2–4.5)	4 (2.5–8.5)	*U* = 85; *p* = 0.254
Latency to drink (s)	44 (12.5–85)	12 (4.5–30)	*U* = 73.5; *p* = 0.106
Latency to drink – latency to contact (s)	42 (9.5–83)	3 (2–25)	*U* = 72; *p* = 0.093
**Neophagia II (Noyes/Y-maze)**			
Latency to contact (s)	40 (20–128.5)	18 (10.5–28)	*U* = 65.5; *p* = 0.051
Latency to eat (s)	217 (147.5–279.5)	110 (77.5–160.5)	*U* = 39; *p* = 0.002**
Latency to eat – latency to contact (s)	108 (82.5–216.5)	93 (36–140)	*U* = 89.5; *p* = 0.340

**p < 0.01. Non-parametric data are presented as median (inter-quartile range) and were analysed with Mann–Whitney *U*-tests. *n* = 15 WT; *n* = 15 *GRM3*^*−/−*^.

*GRM3*^*−/−*^ mice did demonstrate shorter latencies to enter the open arms in the elevated plus maze (*U* = 58; *p* < 0.05; Table 3), and to approach (*U* = 65.5; *p* < 0.05) and eat (*U* = 39.0; *p* < 0.01) the novel food in the second neophagia test compared to controls (Table 6), potentially consistent with reduced anxiety in these animals. A similar, but milder, profile was also evident in the open field test, in which there was a trend for *GRM3*^*−/−*^ mice to be faster to enter the central area of the arena (*t*(28) = 1.89; *p* = 0.07; Table 3). However, no differences were found in either % time in open arms (elevated plus maze, Table 3), or latencies to consume after contact (difference score; latency to eat minus latency to contact; Neophagia tests, Table 6), or total time spent in the central area (open field test, Table 3), which are more widely used measures of anxiety-like behaviours in these tests.

### 3.2 Motor tests

The results of the motor tests are summarised in Table 7, Table 8, Table 9. Observation of the mice in their homecages revealed no noticeable differences in motor function between the groups. However, *GRM2*/3^*−/−*^ mice were significantly impaired in tests assessing motor coordination (Table 7). On the accelerating rotarod, *GRM2/3*^*−/−*^ mice fell from the rod sooner (*t*(30) = 2.33; *p* < 0.05), while it was rotating at a lower speed (*t*(30) = 2.29; *p* < 0.05). This cannot be attributed to differences in body weight (see above). *GRM2/3*^*−/−*^ mice were also impaired on the multiple static rods test of motor coordination, taking longer (i) to turn around on the rod and (ii) to traverse its length (Table 7). Performance in the multiple static rods test was analysed using separate two-way RM-ANOVA for orientation times (OTs) and transit times (TTs). Average OTs and TTs for rods 1–3 were used in each analysis (as values for individual rods were very similar) together with the individual values for rods 4 and 5. OT data were subjected to a log_10_ transformation prior to analysis to ensure that the data satisfied the criteria for parametric analysis. Two-way RM-ANOVA for the transformed OT data revealed an effect of group (*F*_(1,30)_ = 8.03; *p* < 0.01) and an effect of rod (*F*_(2,60)_ = 66.20; *p* < 0.01), plus a group by rod interaction (*F*_(2,60)_ = 7.76; *p* < 0.01). Duncan's multiple pairwise comparisons revealed that the performance of *GRM2/3*^*−/−*^ double KO and WT mice differed significantly on rod 5 (*p* < 0.05). TT data were also log_10_ transformed, and two-way RM-ANOVA again revealed an effect of group (*F*_(1,30)_ = 5.12; *p* < 0.05), an effect of rod (*F*_(2,60)_ = 85.08; *p* < 0.01), and a group by rod interaction (*F*_(2,60)_ = 8.07; *p* < 0.01). Duncan's multiple pairwise comparisons confirmed that, in common with the OT data, the performance of the two groups differed significantly on rod 5 (*p* < 0.05). Overall, the motor tests reveal a relatively mild, but significant, motor deficit in *GRM2/3*^*−/−*^ mice.

**Table 7:** Comparison of *GRM2/3*^*−/−*^ mice and wild-types (WT) in laboratory tests of motor function.

Task & measure	WT	*GRM2/3*^*−/−*^	Statistics
**Horizontal Bar**			
Score/5	5 (5–5)	5 (5–5)	*U* = 194.5; *p* = 0.939
**Accelerating Rotarod**			
Speed at fall (rpm)	19.0 ± 1.4	14.2 ± 1.6	*t*(30) = 2.29; *p* = 0.029*
Latency to fall (s)	47.5 ± 4.3	32.4 ± 4.8	*t*(30) = 2.33; *p* = 0.027*
**Multiple Static Rods**			
**Orientation Time (OT)**			
Average OT for rods 1–3 (s)	4.8 ± 0.7	5.2 ± 0.9	Main effect of group: *F*_(1,30)_ = 8.03; *p* = 0.008**
OT for rod 4 (s)	4.2 ± 0.6	6.2 ± 1.2	Main effect of rod: *F*_(2,60)_ = 66.20; *p* < 0.001**
OT for rod 5 (s)	56.1 ± 18.7	143.1 ± 19.6	Group*rod interaction: *F*_(2,60)_ = 7.76; *p* = 0.001**#
**Transit Time (TT)**			
Average TT for rods 1–3 (s)	9.6 ± 1.7	8.8 ± 1.4	Main effect of group: *F*_(1,30)_ = 5.12; *p* = 0.031*
TT for rod 4 (s)	8.3 ± 0.8	9.6 ± 1.4	Main effect of rod: *F*_(2,60)_ = 85.08; *p* < 0.001**
TT for rod 5 (s)	62.1 ± 17.8	144.3 ± 19.0	Group*rod interaction: *F*_(2,60)_ = 8.07; *p* < 0.001**#
**Body Weight (g)**	28.5 ± 0.3	28.0 ± 0.4	*t* < 1; *p* > 0.20

*p < 0.05; **p < 0.01 Parametric data are presented as mean ± SEM, while non-parametric data are presented as median (inter-quartile range). Horizontal bar data were analysed using a Mann–Whitney *U*-test, and rotarod data using a *t*-test. OT and TT data were analysed using two-way Repeated Measures ANOVA on the log_10_ transformed data. Any significant effects were further investigated using Duncan's multiple pairwise comparisons (#*p* < 0.05). *n* = 18 WT; *n* = 14 *GRM2/3*^*−/−*^.

**Table 8:** Comparison of *GRM2*^*−/−*^ mice and wild-types (WT) in laboratory tests of motor function.

Task & measure	WT	*GRM2*^*−/−*^	Statistics
**Horizontal Bar**			
Score/5	5 (5–5)	5 (4–5)	*U* = 81.5; *p* = 0.305
**Accelerating Rotarod**			
Speed at fall (rpm)	8.3 (5.35–14.6)	7.4 (5.5–11.62)	*U* = 82.0; *p* = 0.383
Latency to fall (s)	27 (20–46)	25 (19–35)	*U* = 53.5; *p* = 0.738
**Multiple Static Rods**			
**Orientation Time (OT)**			
Average OT for rods 1–3 (s)	19.3 ± 7.5	23.4 ± 8.4	Main effect of group: *F* < 1; *p* > 0.80
OT for rod 4 (s)	29.1 ± 17.1	40.8 ± 20.2	Main effect of rod: *F*_(2,54)_ = 18.04; *p* < 0.001**
OT for rod 5 (s)	33.5 ± 16.7	43.9 ± 19.7	Group*rod interaction: *F* < 1; *p* > 0.90
**Transit Time (TT)**			
Average TT for rods 1–3 (s)	35.9 ± 8.7	45.4 ± 10.0	Main effect of group: *F* < 1; *p* > 0.80
TT for rod 4 (s)	32.8 ± 18.5	53.3 ± 15.6	Main effect of rod: *F*_(2,54)_ = 17.58; *p* < 0.001**
TT for rod 5 (s)	86.5 ± 20.6	62.6 ± 19.1	Group*rod interaction: F_(2,54)_ = 2.20; *p* = 0.120
**Body Weight (g)**	29.2 ± 0.7	28.0 ± 0.4	*t*(27) = 1.34; *p* = 0.191

**p < 0.01 Horizontal bar and rotarod tests: parametric data are presented as mean ± SEM and were analysed with *t*-tests. Non-parametric data are presented as median (inter-quartile range) and were analysed with Mann–Whitney *U*-tests. Multiple Static Rods test: data are presented as mean ± SEM. OT and TT data were analysed using two-way Repeated Measures ANOVA on the log_10_ transformed data. *n* = 15 WT; *n* = 14 *GRM2*^*−/−*^.

**Table 9:** Comparison of *GRM3*^*−/−*^ mice and wild-types (WT) in laboratory tests of motor function.

Task & measure	WT	*GRM3*^*−/−*^	Statistics
**Horizontal Bar**			
Score/5	5 (5–5)	5 (5–5)	*U* = 105; *p* = 0.756
**Accelerating Rotarod**			
Speed at fall (rpm)	10.4 (9.03–14.1)	9.8 (7.6–10.7)	*U* = 127.5; *p* = 0.534
Latency to fall (s)	24.6 ± 2.9	22.0 ± 2.8	*t* < 1; *p* > 0.50
**Multiple Static Rods**			
**Orientation Time (OT)**			
Average OT for rods 1–3 (s)	8.7 ± 1.6	5.3 ± 0.5	Main effect of group: *F* < 1; *p* > 0.50
OT for rod 4 (s)	4.9 ± 0.8	5.9 ± 1.0	Main effect of rod: F_(2,56)_ = 4.91; *p* = 0.011**
OT for rod 5 (s)	9.8 ± 1.5	8.5 ± 1.7	Group*rod interaction: *F* < 1; *p* > 0.30
**Transit Time (TT)**			
Average TT for rods 1–3 (s)	21.9 ± 3.6	23.7 ± 3.1	Main effect of group: *F* < 1; *p* > 0.80
TT for rod 4 (s)	21.3 ± 4.2	14.8 ± 2.1	Main effect of rod: *F*_(2,56)_ = 8.92; *p* < 0.001**
TT for rod 5 (s)	34.5 ± 6.8	46.4 ± 14.4	Group*rod interaction: *F*_(2,56)_ = 113; *p* = 0.331
**Body Weight (g)**	27.8 ± 0.4	27.6 ± 0.4	*t* < 1; *p* > 0.70

**p < 0.01. Horizontal bar and rotarod tests: parametric data are presented as mean ± SEM and were analysed with *t*-tests. Non-parametric data are presented as median (inter-quartile range) and were analysed with Mann–Whitney *U*-tests. Multiple Static Rods test: data are presented as mean ± SEM. OT and TT data were analysed using two-way Repeated Measures ANOVA on the log_10_ transformed data. *n* = 15 WT; *n* = 15 *GRM3*^*−/−*^.

In contrast, there were no differences between either *GRM2*^*−/−*^ or *GRM3*^*−/−*^ mice and their WT control mice on any of the motor tests (Table 8, Table 9).

### 3.3 Spontaneous locomotor activity

**Fig. 1:**
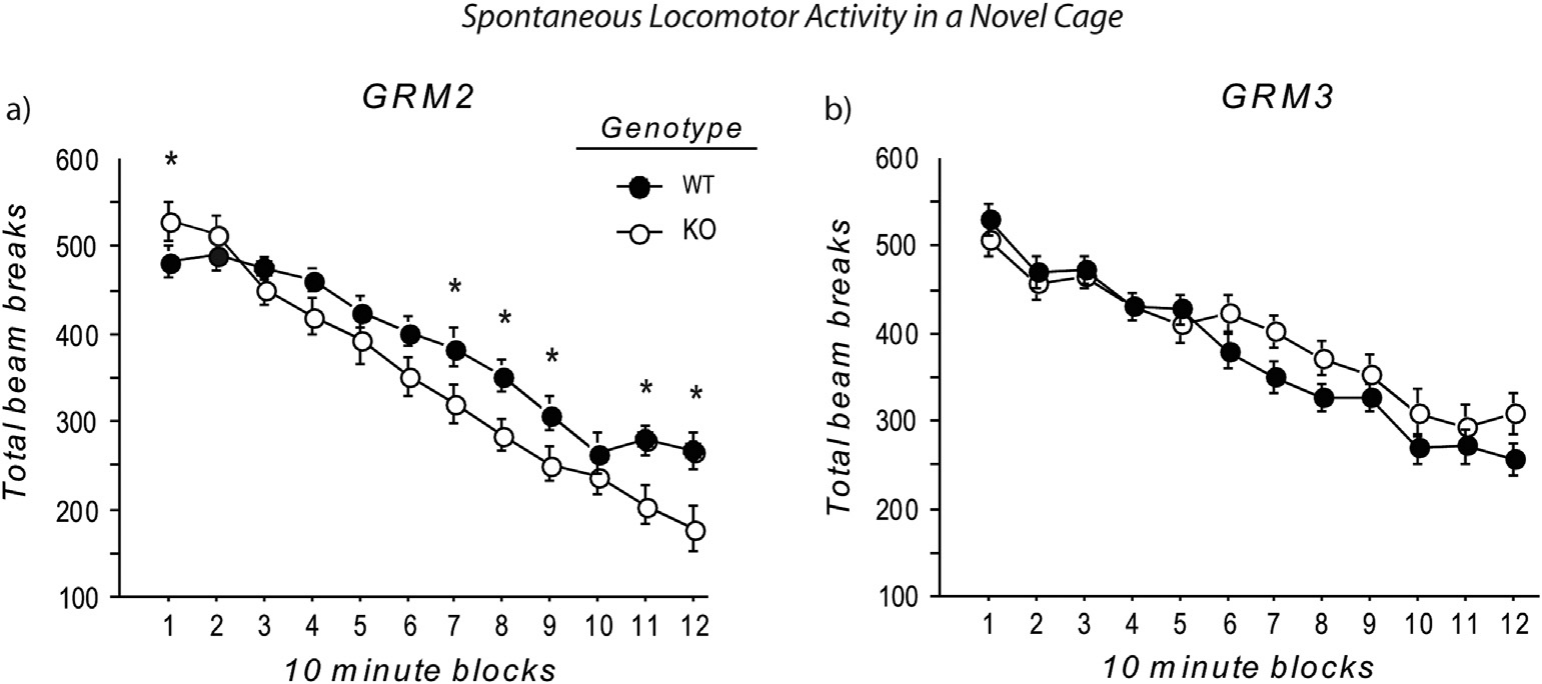
Spontaneous locomotor activity during a two-hour period in a novel home cage environment. (a) *GRM2*^−/−^ mice were initially hyperactive but then their activity levels fell below those of the WT mice for most of the 2nd hour of recording. *n* = 15 WT; *n* = 14 *GRM2*^*−/−*^. (b) *GRM3*^*−/−*^ mice showed similar activity levels compared to WT controls, although the temporal profile across the session was subtly altered. *n* = 15 WT; *n* = 15 *GRM3*^*−/−*^. Data are mean beam breaks ± SEM for each block of 10 min, and were analysed using a two way, repeated measures ANOVA, followed by analysis of simple main effects. Asterisks indicate statistical significance at *p* < 0.05 from analysis of simple main effects.

We have reported previously that spontaneous locomotor activity was reduced in the *GRM2/3*^*−/−*^ mice in photocell activity cages (Lyon et al., 2011). Assessment of *GRM2*^*−/−*^ mice in the same apparatus revealed an interesting pattern of results (Fig. 1a). The *GRM2*^*−/−*^ mice were initially hyperactive but then their activity levels fell below those of the WT mice such that the KOs were hypoactive during the second hour of recording. ANOVA revealed a significant genotype × block interaction (*F*_(11,297)_ = 2.61; *p* < 0.01). Simple main effects analysis revealed significant hyperactivity in the KO mice during the first 10 min time bin, but then hypoactivity in bins 7–9, 11 and 12 (60–120 min) (all *p* < 0.05).

A different pattern of results was evident in *GRM3*^*−/−*^ mice (Fig. 1b). Overall, *GRM3*^*−/−*^ mice did not differ significantly in activity levels from their WT littermates during the 2 h test (main effect of genotype: *F* < 1; *p* > 0.50). However, there was a significant genotype by time bin interaction (*F*_(11,308)_ = 2.07; *p* < 0.05), which seemed to reflect a slower decrease in activity levels in the *GRM3*^*−/−*^ mice compared to the controls during the second hour of testing, although simple main effects analysis showed that there were no significant effects of genotype for any time bin individually (all *p* > 0.05).

### 3.4 Cognitive tests

#### 3.4.1 Spontaneous spatial novelty preference task

**Fig. 2:**
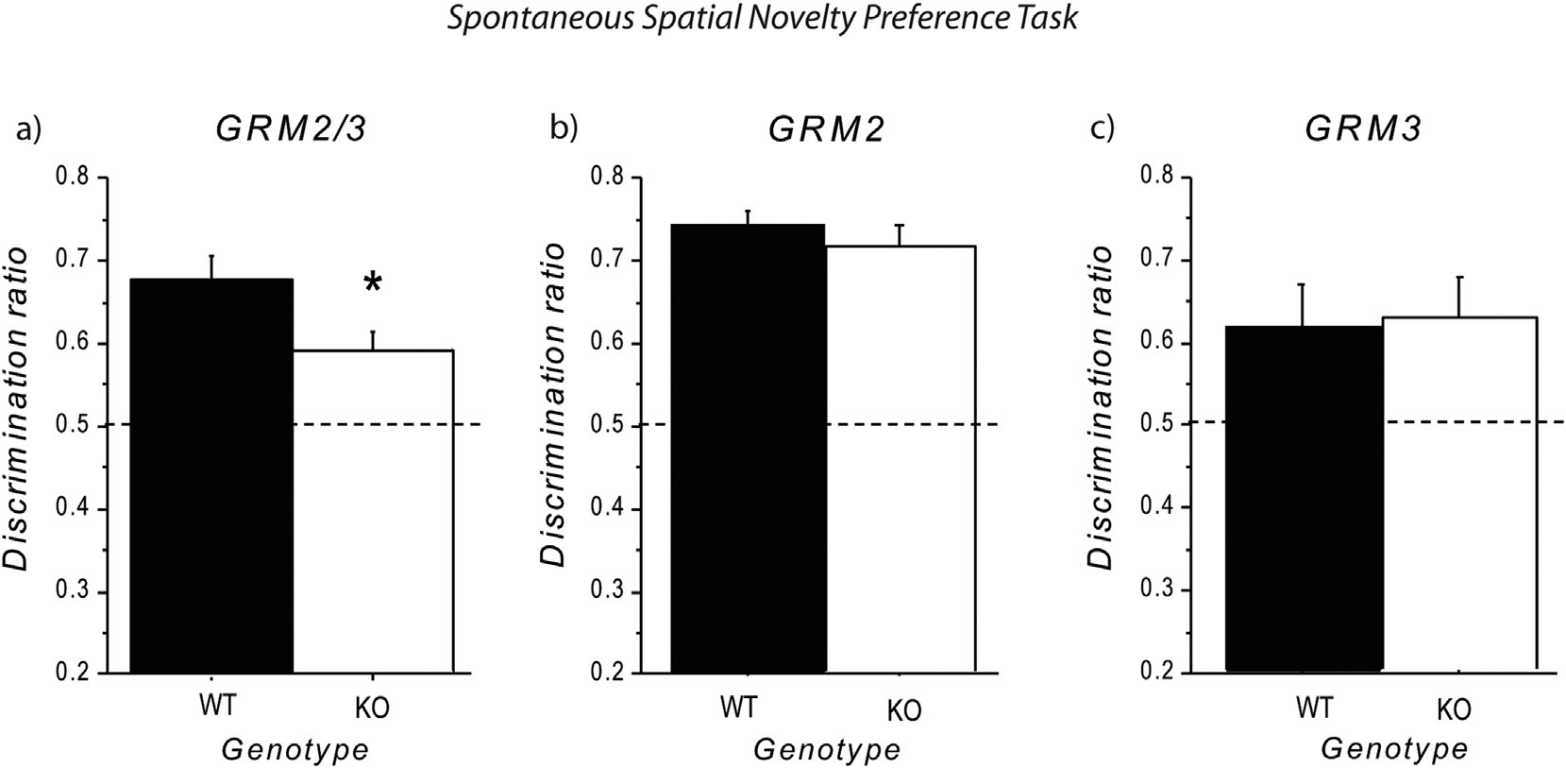
Spontaneous spatial novelty preference task. (a) *GRM2/3*^*−/−*^ mice showed reduced spatial novelty preference compared with WT animals. *GRM2*^*−/−*^ (b) and *GRM3*^*−/−*^ (c) mice did not differ from WT controls. Data shown are mean discrimination ratios for the time spent in the arms during the test phase [novel arm/(novel + other arm)] ± SEM. A discrimination ratio of 0.5 reflects chance performance. Asterisk indicates a statistically significant difference between groups (*t*-test at *p* < 0.05). *n* = 11 WT; *n* = 11 *GRM2/3*^*−/−*^; *n* = 15 WT; *n* = 14 *GRM2*^*−/−*^; *n* = 15 WT; *n* = 15 *GRM3*^*−/−*^.

In agreement with our previous study (Lyon et al., 2011), we again found that *GRM2/3*^*−/−*^ mice exhibited impaired short-term spatial memory on the exploratory driven, spatial novelty preference task (Fig. 2a). During the exposure phase, both WT and *GRM2/3*^*−/−*^ mice spent a similar amount of time exploring the “other” (to-be-familiar) arm (WT = 120.8 ± 8.4, *GRM2/3*^*−/−*^ mice = 114.4 ± 5.2 s; *t* < 1; *p* > 0.50). However, *GRM2/3*^*−/−*^ mice made significantly fewer arm entries (start and other arms combined) during the exposure phase (WT = 44.3 ± 2.2, *GRM2/3*^*−/−*^ mice = 30.5 ± 2.5; *t*(20) = 4.13; *p* < 0.01), thus confirming a hypoactive phenotype in these mice (Lyon et al., 2011). During the subsequent test phase, both groups showed a significant preference for the previously unvisited, “novel” arm over the now familiar, “other” arm. A discrimination ratio was calculated [(novel arm)/(novel + other arm)] for time spent in arms during the test phase, and statistical analysis revealed that the preference for the novel arm was significantly above chance for both genotypes (one group *t*-tests conducted against a single value of 0.5 which corresponds to chance performance; *p*'s < 0.01). Importantly, the WT mice displayed a significantly greater novelty preference than the *GRM2/3*^*−/−*^ mice (effect of genotype-*t*(20) = 2.23; *p* < 0.05). *GRM2/3*^*−/−*^ mice also made significantly fewer arms entries (all three arms combined) during the test phase (WT = 17.2 ± 1.1; *GRM2/3*^*−/−*^ mice = 12.1 ± 0.9; *t*(20) = 3.46; *p* < 0.01).

There was no difference between *GRM2*^*−/−*^ mice and their WT controls on the spatial novelty preference task (Fig. 2b). During the exposure phase, time exploring the “other” arm did not differ between WT and *GRM2*^*−/−*^ mice (WT = 85.1 ± 5.3 s, *GRM2*^*−/−*^ mice = 93.8 ± 3.9 s; *t*(27) = 1.30; *p* = 0.20). A similar number of arm entries (start and other arms combined) was also evident during the exposure phase (WT = 27.6 ± 1.3 s, *GRM2*^*−/−*^ mice = 28.1 ± 1.6 s; *t* < 1; *p* > 0.80). During the test phase, both groups showed a significant preference for the novel arm (one group *t*-tests conducted against a single value of 0.5 which corresponds to chance performance; *p*'s < 0.01), and this did not differ between the genotypes (*t* < 1; *p* > 0.30). There was no effect of genotype on number of arm entries (all three arms combined) made during the test phase (WT = 13.5 ± 0.6, *GRM2*^*−/−*^ mice = 14.5 ± 0.8; *t* < 1; *p* > 0.30).

Similarly, the *GRM3*^*−/−*^ mice did not differ significantly from their WT littermates (Fig. 2c) during the exposure phase in terms of time exploring the “other” arm (WT = 100.3 ± 6.5 s, *GRM3*^*−/−*^ = 100.5 ± 6.8 s; *t* < 1; *p* > 0.70), and total number of arm entries (WT = 24.7 ± 7.5 s, *GRM3*^*−/−*^ = 30.9 ± 9.5 s; *t*(28) = 1.95; *p* = 0.06). During the test phase, a significant preference for the novel arm (one group *t*-tests conducted against a single value of 0.5 which corresponds to chance performance; *p*'s < 0.01) was again shown by both groups and the discrimination ratio for time in arms did not differ between genotypes (*t* < 1; *p* > 0.80). The number of arm entries during the test phase was also comparable between genotypes (WT = 13.7 ± 3.1, *GRM3*^*−/−*^ = 13.9 ± 2.9; *t* < 1; *p* > 0.80).

#### 3.4.2 Spatial working memory on the elevated T-maze

Spatial working memory was assessed during non-matching to place testing (rewarded alternation) on the elevated T-maze. We have previously shown that *GRM2/3*^*−/−*^ mice display a robust and enduring impairment on this task (Lyon et al., 2011). In addition, these double KO mice exhibited shorter latencies to complete both the sample and the choice runs of the task.

**Fig. 3:**
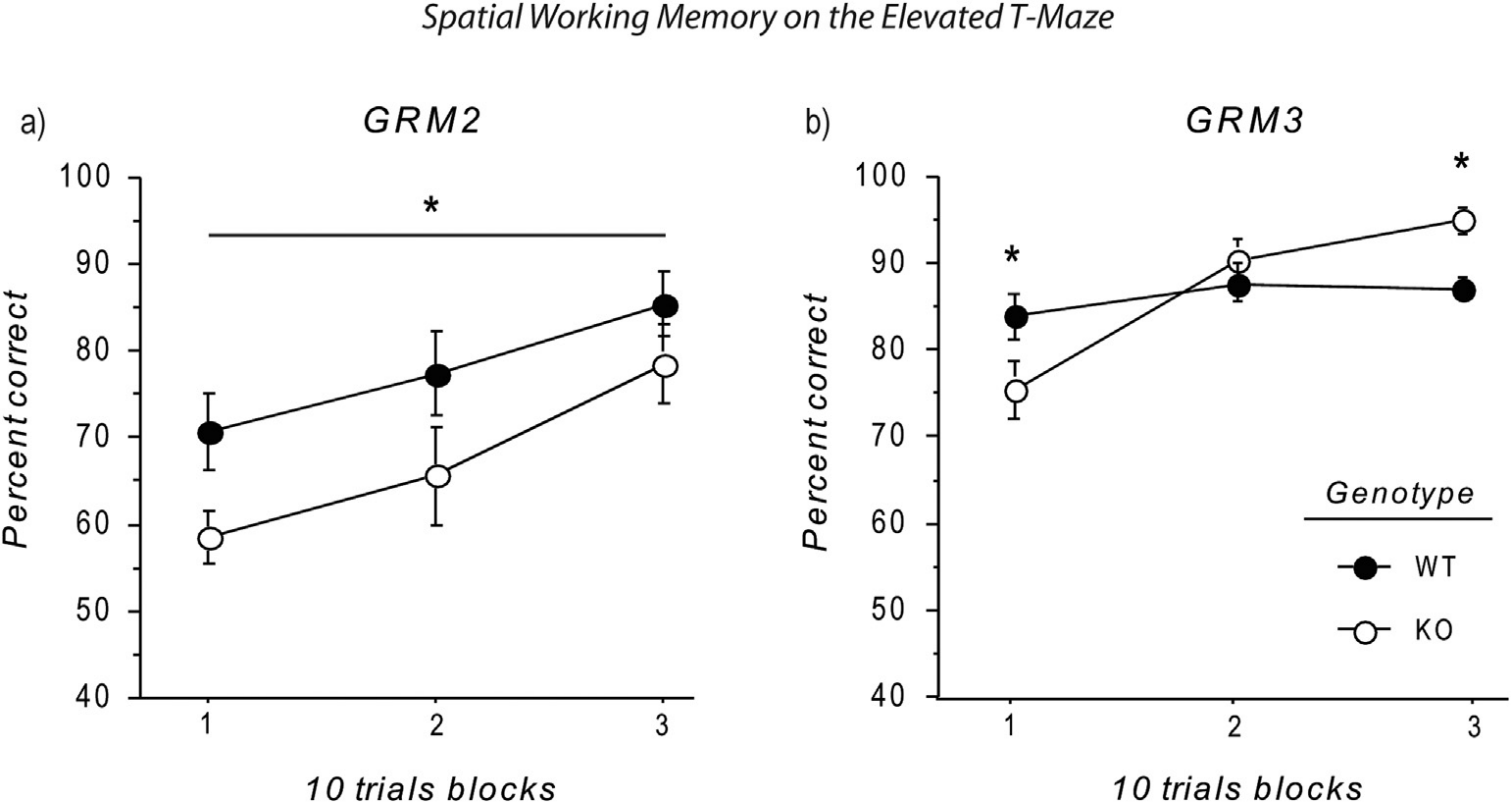
Spatial working memory on the elevated T-maze. (a) *GRM2*^*−/−*^ mice were persistently impaired in the appetitive T-maze rewarded alternation task compared with WTs. *n* = 15 WT; *n* = 14 *GRM2*^*−/−*^ (significant overall main effect of genotype; *p* < 0.05). (b) *GRM3*^−/−^ mice showed poorer performance during the first 10 trial block but better performance during the last block compared to littermate controls. *n* = 15 WT; *n* = 15 *GRM3*^*−/−*^. Data shown are mean percent correct responses (±SEM) for each block of 10 trials, and were analysed using a two way, repeated measures ANOVA, followed by analysis of simple main effects if a significant genotype by block interaction was present (i.e. (b) GRM3). Asterisks indicate statistical significance at *p* < 0.05 between the genotypes.

Single *GRM2*^*−/−*^ mice were impaired in terms of choice accuracy on the spatial working memory T-maze task across the three blocks of testing (main effect of genotype – *F*_(1,27)_ = 4.24; *p* < 0.05; Fig. 3a). No genotype by block interaction was evident (*F* < 1; *p* > 0.60). Furthermore, they also had shorter run latencies than the WT controls on both the sample (WT = 14.9 ± 2.9 s, *GRM2*^*−/−*^ mice = 3.6 ± 0.3 s; main effect of genotype – *F*_(1,27)_ = 5.36; *p* < 0.05) and choice runs (WT = 11.7 ± 2.0 s, *GRM2*^*−/−*^ mice = 3.4 ± 0.5 s; main effect of genotype – *F*_(1,27)_ = 5.45; *p* < 0.05).

*GRM3*^*−/−*^ mice were impaired on the first block of spatial working memory testing, but improved across the three blocks such that they were actually more accurate than WT by the end of testing (Fig. 3b). There was a significant genotype by block interaction (*F*_(2,56)_ = 6.28; *p* < 0.01). Simple main effects analysis revealed that *GRM3*^*−/−*^ mice were impaired compared to WT controls during the first 10 trial block, but exhibited better performance during the last block compared to their littermate controls (all *p* < 0.05). *GRM3* deletion had no effect on the run latencies for either the sample (*F* < 1; *p* > 0.90) or the choice runs (*F* < 1; *p* > 0.80).

#### 3.4.3 Appetitively motivated spatial reference memory on the elevated Y-maze

**Fig. 4:**
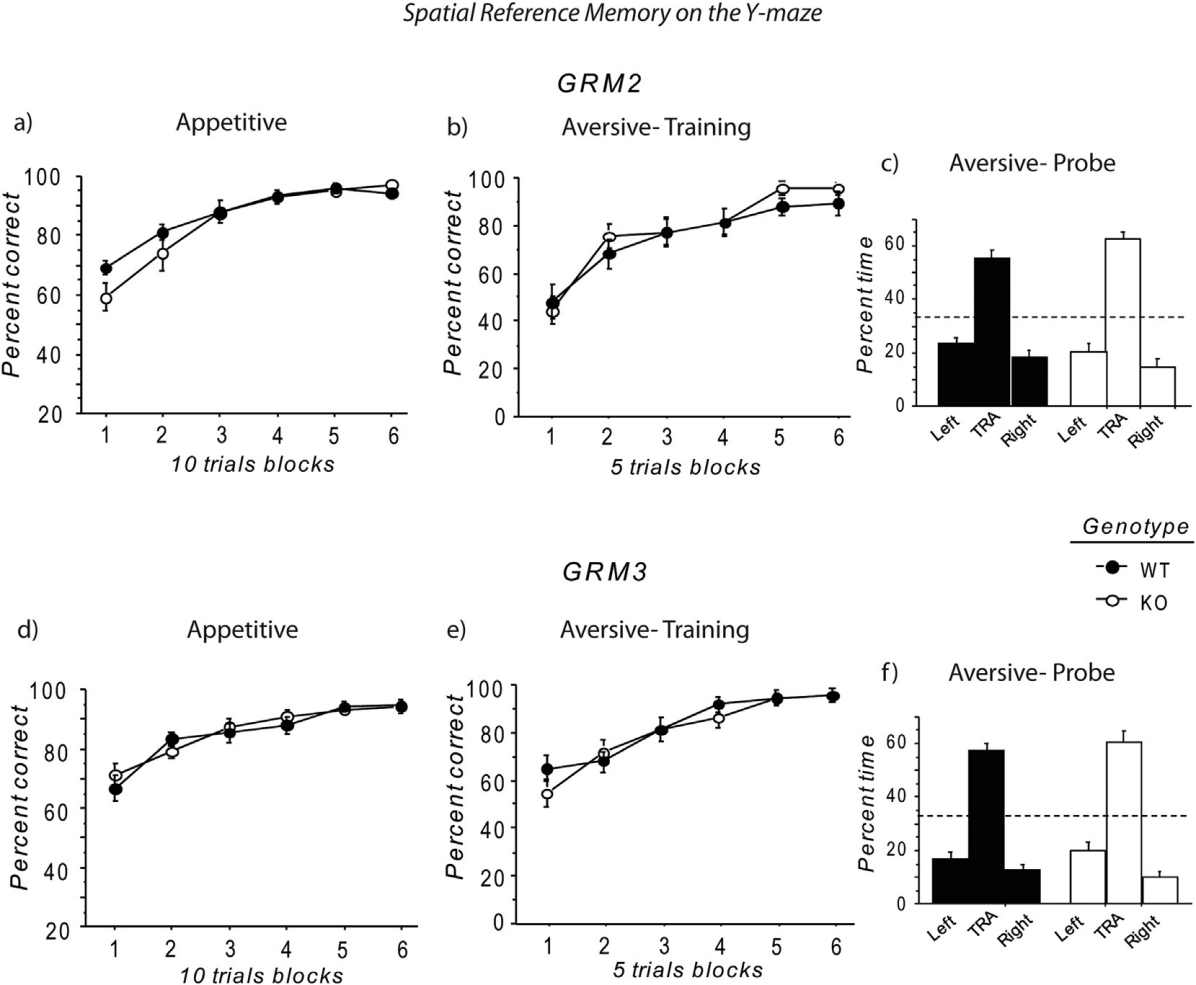
Appetitive and aversive spatial reference memory tasks. (a) *GRM2*^−/−^ mice acquired the appetitive spatial reference memory Y-maze task as well as the WT controls. Data shown are mean percent correct responses (±SEM) for each block of 10 trials. (b) *GRM2*^−/−^ mice also learned the aversive, swimming spatial reference memory Y-maze task at the same rate as WT mice. Data shown are mean percent correct responses (±SEM) for each block of 5 trials. (c) In the probe test on day 7 of the aversive, swimming Y-maze task, *GRM2*^−/−^ and WT mice spent an equal proportion of time searching in the arm that had previously held the platform (TRA). Thirty three percent time in an arm reflects chance performance (broken line). (d) *GRM3*^−/−^ mice displayed similar acquisition of the appetitive spatial reference memory Y-maze task as the WT controls. Data shown are mean percent correct responses (±SEM) for each block of 10 trials. (e) *GRM3*^−/−^ mice also learned the aversive, swimming spatial reference memory Y-maze task at the same rate as WT mice. Data shown are mean percent correct responses (±SEM) for each block of 5 trials. (f) In the probe test on day 7 of the aversive, swimming Y-maze task, *GRM3*^−/−^ and WT mice spent an equal proportion of time searching in the arm that had previously held the platform (TRA). Thirty three percent time in an arm reflects chance performance (broken line). Acquisition data were analysed using a two way, repeated measures ANOVA. Time spent in the target arm during the probe test at the end of training for the two groups were compared using a *t*-test. Order of testing and the rooms in which the tests were performed were fully counterbalanced. *n* = 15 WT; *n* = 14 *GRM2*^*−/−*^; *n* = 15 WT; *n* = 15 *GRM3*^*−/−*^ mice.

We reported previously that *GRM2/3*^*−/−*^ mice were impaired on an appetitively motivated, spatial reference memory task on the elevated Y-maze (Lyon et al., 2011). Here, neither the single *GRM2*^*−/−*^ nor single *GRM3*^*−/−*^ mice were impaired on this task (Fig. 4a, d). Although there was some suggestion that the *GRM2*^*−/−*^ mice performed significantly less accurately on the first block of testing, ANOVA revealed neither a main effect of genotype (*F* < 1; *p* > 0.40), nor a significant genotype by block interaction (*F*_(5,125)_ = 2.08; *p* = 0.07), although there was a significant main effect of block (*F*_(5,125)_ = 55.71; *p* < 0.01; Fig. 4a). Because the test order by block interaction was also significant (*F*_(5,125)_ = 3.32; *p* < 0.01), a further analysis was performed, restricted to just those animals that were experimentally naïve with respect to the appetitive Y-maze (i.e. those mice that had not previously been trained on the aversive, swim-escape task). A similar result was obtained. Again, there was a main effect of block (*F*_(5,60)_ = 33,91; *p* < 0.01), but neither a main effect of genotype (*F* < 1; *p* > 0.50), nor a genotype by block interaction (*F*_(5,60)_ = 1.11; *p* = 0.37).

For the *GRM3*^*−/−*^ mice, there was no sign of any spatial reference memory impairment. ANOVA revealed a main effect of block (*F*_(5,130)_ = 30.93; *p* < 0.01), and a test order by block interaction (*F*_(5,130)_ = 3.05; *p* < 0.05), but no effect of genotype (*F* < 1; *p* > 0.80), nor genotype by block interaction (*F* < 1; *p* > 0.50; Fig. 4d). The same result was obtained when the analysis excluded those mice that had previously been trained on the aversive, swim-escape task (main effect of block – *F*_(5,70)_ = 13.28; *p* < 0.01; no effect of genotype – *F*_(1,14)_ = 2.29; *p* = 0.15; no genotype by block interaction – *F*_(5,70)_ = 1.31; *p* = 0.27).

The performance of the animals on the first block of testing in these experiments was better than expected, and better than we have experienced previously (e.g. Bannerman et al., 2012, Lyon et al., 2011). The reason is not immediately obvious. It did not reflect an innate bias to one of the arms of the maze. Target arm allocation between the three arms of the maze was counterbalanced within each genotype, and analysis of the data with target arm included as a between subjects factor generated no main effect of target arm (*GRM3*^*−/−*^ cohort: *F*_(2,24)_ < 1; *p* > 0.60; *GRM2*^*−/−*^ cohort: *F*_(2,23)_ < 1; *p* > 0.80) nor genotype by target arm interactions (*GRM3*^*−/−*^ cohort: *F*_(2,24)_ < 1; *p* > 0.90; *GRM2*^*−/−*^ cohort: *F*_(2,23)_ < 1; *p* > 0.60). It is also not the case that the mice were solving the task by smelling the food reward. During the sixth block of Y-maze testing the milk reward was only delivered after the mice had made a choice (post-choice baiting). Performance remained at a high level on these trials, confirming that the mice were not solving the task using the smell of the reward. Furthermore, the entire maze was rotated periodically to prevent the mice from using intramaze cues. Initially high performance levels also didn't appear to reflect any effect of the prior training on the aversive swim/escape Y-maze task, as performance levels in the two sub-populations of mice (Y-maze naïve vs. Y-maze experienced) were very similar. Thus, it seems most likely that there was within-session learning during the first 10 trials of training. Importantly, analysis of just the very first training trial for each animal in the two studies was not significantly above chance (62.7% correct; binomial test: *p* > 0.05).

#### 3.4.4 Aversively motivated spatial reference memory in the Y-maze

Both *GRM2*^*−/−*^ and *GRM3*^*−/−*^ mice acquired the aversive, swim/escape Y-maze reference memory task as well as their respective controls, consistent with our previous study in the *GRM2/3*^*−/−*^ mice which had also revealed no impairment (Lyon et al., 2011). *GRM2*^*−/−*^ mice displayed normal choice performance during training (main effect of genotype – *F* < 1; *p* > 0.40; genotype by block interaction – *F* < 1; *p* > 0.70; Fig. 4b), and showed an equivalent preference for the target arm during the 30 s probe test at the end of testing (genotype comparison; *t*(27) = 1.54; *p* = 0.14; % time in target arm: WT = 55.5 ± 3.2, one group *t*-test conducted against a single value of 33.3% which corresponds to chance performance; *t*(14) = 6.91; *p* < 0.01; *GRM2*^*−/−*^ mice = 62.2 ± 2.9, one group *t*-test conducted against a single value of 33.3% which corresponds to chance performance; *t*(13) = 9.96; *p* < 0.01; Fig. 4c). The same result was obtained when mice that had already performed the appetitive task were excluded (data not shown).

Likewise, *GRM3*^*−/−*^ mice were normal during acquisition (main effect of genotype – *F* < 1; *p* > 0.50; genotype by block interaction – *F* < 1; *p* > 0.50; Fig. 4e), and spent a similar amount of time in the arm that had previously contained the platform during the probe test (genotype comparison; *t* < 1; *p* > 0.50; % time in target arm: WT = 57.3 ± 2.7, one group *t*-test conducted against a single value of 33.3% which corresponds to chance performance; *t*(14) = 8.99; *p* < 0.01; *GRM3*^*−/−*^ mice = 60.6 ± 4.0, one group *t*-test conducted against a single value of 33.3% which corresponds to chance performance; *t*(14) = 6.86; *p* < 0.01; Fig. 4f). Again, this was true irrespective of whether the analysis was limited to Y-maze naïve subjects or also included mice that had already completed the appetitive task (data not shown).

## 4 Discussion

In the present series of experiments we have extended our behavioural characterisation of *GRM2/3*^*−/−*^ mice (Lyon et al., 2011) and, in addition, investigated the behavioural phenotype of single *GRM2*^*−/−*^ and *GRM3*^*−/−*^ mice to extend earlier work by other groups (Fell et al., 2011, Fujioka et al., 2014, Lainiola et al., 2014, Linden et al., 2005, Morishima et al., 2005). The results are summarised in Table 10. We found no consistent effect on anxiety in either the double or single KO mice. The *GRM2/3*^*−/−*^ mice were impaired on a novelty preference test assessing short-term spatial memory, but this phenotype was absent in both *GRM2*^*−/−*^ and *GRM3*^*−/−*^ mice. Similarly, while we have shown previously that *GRM2/3*^*−/−*^ mice are impaired at acquiring an appetitively (but not aversively) motivated spatial reference memory Y-maze task (Lyon et al., 2011), we found no such impairment in either of the single KO lines. In contrast, spatial working memory (rewarded alternation) testing on the elevated T-maze revealed a clear impairment in the *GRM2*^*−/−*^ mice throughout testing (although again the effect was not as big as in the double KOs), whereas *GRM3*^*−/−*^ mice exhibited a biphasic effect (initially impaired but performing better than controls by the end of training). A biphasic effect on activity levels was also seen for the *GRM2*^*−/−*^ mice during testing in photocell activity cages. The *GRM2/3*^*−/−*^ mice (but neither single KO line) were also impaired on tests of motor co-ordination, demonstrating that their behavioural phenotype extends beyond the domain of hippocampus-dependent spatial memory (see also Morishima et al., 2005). These findings have implications for the roles which the group II metabotropic glutamate receptors, individually and collectively, play in anxiety, cognition, and their interaction.

**Table 10:** Summary.

Behavioural domains	*GRM2/3*^*−/−*^	*GRM2*^*−/−*^	*GRM3*^*−/−*^
Emotionality (Anxiety)	=	=	=
Motor Co-ordination Tests	↓	=	=
Spontaneous Locomotor Activity	↓*	**↓↑**	#
Spatial Novelty Preference	↓	**=**	=
Spatial Working Memory (Appetitive)	↓↓*	**↓**	↓↑
Spatial Reference Memory (Appetitive)	↓*	**=**	=
Spatial Reference Memory (Aversive)	=*	**=**	=

=: not significantly different from their respective wild-types (WT) controls; ↓: slightly impaired compared to their respective WT controls; ↓↓: considerably impaired compared to their respective WT controls; ↑: better performance compared to their respective WT controls; ↓↑: bi-phasic response. #: There was no significant main effect of genotype but a significant genotype by time bin interaction. However, there were no significant genotype differences in any individual time bin. *: Published in Lyon et al. (2011).

### 4.1 A stronger behavioural phenotype in *GRM2/3^−/−^* mice than in either *GRM2^−/−^* or *GRM3^−/−^* mice

A key finding from these studies is that the behavioural phenotype in the double KO mice is more pronounced than the phenotype in either of the single lines. In the present study this is evident in the spatial novelty preference test for example. There was no effect on short-term spatial memory in this task in either single line (see also Lainiola et al., 2014 for *GRM3*^*−/−*^ mice), whereas there was a clear deficit in the double KOs (see also Lyon et al., 2011). Similarly, whereas *GRM2/3*^*−/−*^ mice were impaired on tests of motor coordination such as the accelerating rotarod, there was no sign of a phenotype in the single KO lines in our studies. The absence of a deficit on the accelerating rotarod test in the *GRM2*^*−/−*^ mice in the present study is at odds with a previous paper reporting a motor deficit in these animals (Morishima et al., 2005), although our demonstration of impaired motor performance in the *GRM2/3*^*−/−*^ mice is at least consistent with a role for Group II metabotropic receptors in this behaviour. We cannot at this stage determine whether this impairment in our *GRM2/3*^*−/−*^ mice is due to a fundamental problem with motor function (e.g. motor coordination), or whether the deficit reflects an altered reaction to the novelty and/or arousal-inducing properties of the task in the KO mice (see Section 4.3 below for further discussion of a potential role for these receptors at the interface between arousal and behavioural performance). The latter possibility could potentially explain the different outcomes with the *GRM2*^*−/−*^ mice reported here and elsewhere (Morishima et al., 2005). Future studies could examine rotarod performance over repeated testing sessions in order to examine motor learning, although it is worth pointing out that the previously reported deficit in the *GRM2*^*−/−*^ mice on the rotarod test was present throughout repeated testing, and both WT and KOs appeared to improve their performance at a similar rate (Morishima et al., 2005).

Furthermore, whereas we have shown previously that *GRM2/3*^*−/−*^ mice are robustly impaired on appetitively motivated tests of long-term, associative spatial memory (e.g. spatial reference memory on the Y-maze (Lyon et al., 2011)), in the present study we found no such effects in either *GRM2*^*−/−*^ or *GRM3*^*−/−*^ mice. Even when phenotypes were evident in the *GRM2*^*−/−*^ and *GRM3*^*−/−*^ mice (e.g. appetitively motivated spatial working memory on the elevated T-maze), the magnitude of the effect was much smaller than that seen with the *GRM2/3*^*−/−*^ mice (see Lyon et al., 2011).

These results suggest that there is some redundancy of function between the two group II metabotropic glutamate receptor subtypes. It is also possible that there are compensatory changes in the respective single lines which mask the effects of genetically ablating one receptor subtype. For example, Lyon et al. (2008) showed that mRNA expression of the remaining group II receptor subtype is upregulated in the dentate gyrus subfield of the hippocampus of *GRM2*^*−/−*^ and *GRM3*^*−/−*^ mice. Such changes may ameliorate behavioural phenotypes in the single lines. Moreover, recent data show that striatal dopamine is increased in *GRM2/3*^*−/−*^ mice but not in *GRM2*^*−/−*^ or *GRM3*^*−/−*^ mice (Lane et al., 2013), and it is tempting to speculate that this neurochemical difference may, at least in part, constitute a correlate of the emergent behavioural phenotype seen here in the double KO mice.

Of course it is also important to point out that although there is some similarity and overlap between the expression profiles for GRM2 and GRM3, there are also considerable differences in the regional, cellular and sub-cellular distributions of these two receptor subtypes (Ferraguti and Shigemoto, 2006, Harrison et al., 2008, Spooren et al., 2003). This could explain some of the differences in the phenotypes of the single *GRM2*^*−/−*^ and *GRM3*^*−/−*^ mice, but it also begs the question as to how these distinct expression profiles could result in additive effects in behaviour (and possible redundancy of function), and how they could support compensatory mechanisms leading to the rescue of behavioural phenotypes in the single KO lines. The complex behaviours studied here are likely to be supported by neural circuits and networks encompassing a number of brain regions, and it is possible that GRM2 and GRM3 could contribute to the performance of a particular behaviour by acting at different loci. Moreover, it is not inconceivable that an increase in the expression of one receptor subtype at one locus could compensate for the absence of the other at a different site. However, it is also possible that these compensatory changes could occur in a brain region where both receptor subtypes are expressed such as the hippocampus (Lyon et al., 2008). In this regard it is worth pointing out that many of the behaviours that we investigated in the present study, including tests of both spatial memory and anxiety, depend on the hippocampus (see Bannerman et al., 2004 for review).

### 4.2 Knockout of group II metabotropic glutamate receptors has no consistent effect on anxiety

In agreement with previous reports (Fell et al., 2011, Fujioka et al., 2014, Linden et al., 2005, Morishima et al., 2005), we found no consistent evidence for altered anxiety in mice lacking either mGlu2 or mGlu3 alone, suggesting that deletion of just one of the two receptor subtypes is not sufficient to produce robust effects on anxiety-like behaviours. One possible explanation for these null results is either the compensatory changes and/or redundancy of function between these two homologous receptor subtypes in the single KO lines, as discussed in the previous paragraph. A main aim of the present study therefore was to address this issue by assessing anxiety in the *GRM2/3*^*−/−*^ mice.

We assessed anxiety in *GRM2/3*^*−/−*^ mice using several different ethological, unconditioned tests of anxiety which generate an approach avoidance conflict (Barkus et al., 2010, Gray and McNaughton, 2000). We found no effect on anxiety on the elevated plus maze, open field, black & white alley or neophagia (novelty suppressed feeding) tests. This suggests that previous failures to see effects on anxiety with the single group II mGlu KO lines are not simply due to compensatory changes and/or redundancy of function between mGlu2 and mGlu3 in these lines.

Notably, these null results for anxiety in the KO mice contrast with numerous studies which have reported anxiolytic effects with mGlu2/3 agonists (Dunayevich et al., 2008, Grillon et al., 2003, Helton et al., 1998, Linden et al., 2005, Schoepp et al., 2003, Shekhar and Keim, 2000). Importantly, these anxiolytic effects of mGlu2/3 agonists have been observed in both human and rodent studies, and so the lack of an anxiety phenotype in the KO mice is unlikely to be due simply to species differences. Furthermore, the anxiolytic effects of the group II agonists are absent or reduced in single *GRM2*^*−/−*^ and *GRM3*^*−/−*^ mice (Linden et al., 2005), confirming that the drugs are indeed acting through both of the group II metabotropic glutamate receptor subtypes to reduce anxiety.

There are a number of possible explanations for the lack of anxiety phenotypes in the present study despite the clear anxiolytic effects of the group II metabotropic agonists. It may simply be that under the conditions experienced by the mice in these anxiety tests, endogenous glutamate acting on group II metabotropic receptors contributes little to these behaviours, which would be consistent with more recent studies which have failed to see effects of an mGlu2/3 antagonist on anxiety tests like the elevated plus maze (Bespalov et al., 2008).

A related point concerns the inherent variability that is often observed in such ethological, unconditioned anxiety tests (e.g. note the variability in the performance levels of the three WT cohorts in our study), and the possibility that this could lead to “ceiling” or “floor” effects which could mask any genotype effects. For example, in the present studies, WT performance levels on the elevated plus maze varied quite considerably from one cohort to the next (e.g. percent time in open arms ranged from 7.4 to 26.4%). This is an important caveat and we cannot exclude the possibility that fluctuations in WT performance, not only make comparisons across genotypes difficult, but could also obscure genotypic differences from WT controls. However, it is worth pointing out that we saw no differences between the three separate KO lines and their respective WT controls across a number of different anxiety tests, with very different sensorimotor and motivational demands, and with a varying range of performance (anxiety) levels in the control animals. Furthermore, the lack of an anxiety phenotype in both of the single lines (*GRM2*^*−/−*^ and *GRM3*^*−/−*^ mice) is in agreement with previously published results (Fell et al., 2011, Fujioka et al., 2014, Linden et al., 2005, Morishima et al., 2005). For the *GRM2/3*^*−/−*^ mice, the absence of an anxiety phenotype was seen in several experiments in which the WT levels of performance would likely have allowed either increases or decreases in anxiety to be observed (e.g. the percent time in the open arms of the elevated plus maze for these WT controls was 26.4%; see Table 1). Nevertheless, we cannot of course exclude the possibility that on any given test, a different level of performance in the WT controls could have revealed a genotypic difference.

Alternatively, group II mGlu receptors could have an indirect or modulatory role on these emotional behaviours, with the potential for both anxiety promoting and anxiety reducing effects. In genetically modified mice lacking these receptors, the balance between anxiolytic and anxiogenic phenotypes may be maintained, but the flexibility to respond to further perturbations to the system (e.g. following drug administration) may be lost. Of course we also cannot rule out the possibility that developmental adaptations may occur in KO mice which could compensate in adulthood and ameliorate any potential effects on anxiety.

In contrast to our results, increased anxiety in both the open field and elevated plus maze has been reported in a sub strain of Wistar rats (B&K:Wi) which express substantially reduced levels of mGlu2 protein (Ceolin et al., 2011). It is possible that species differences (rat vs. mouse) could be an important factor. Mice may experience much higher levels of stress and arousal when being handled and assessed on tests of anxiety compared to rats, and this could subtly alter the balance between anxiety promoting and anxiety reducing effects, resulting in a different phenotype following mGlu2 ablation in the two species. However, further studies are needed to verify whether the anxiogenic phenotype can be specifically ascribed directly to mGlu2 deficiency in these B&K:Wi rats.

It is also worth noting that we did see some phenotypic differences in the *GRM3*^*−/−*^ mice on the anxiety tests. They were faster to enter the open arms of the elevated plus maze and, to approach and eat the food in the second neophagia test. There was also a trend for them to be faster to enter the central area of the open field. Notably, however, there were no group differences on the more commonly used measures of anxiety in these tests (e.g. percent time in open arms of the elevated plus maze or in the central area of the open field, latency to eat minus latency to contact in the food neophagia test: Barkus et al., 2010, Gray and McNaughton, 2000). In a recent study, Fujioka et al. (2014) also reported increased activity levels in *GRM3*^*−/−*^ mice (e.g. in the open field and in the dark compartment of the light/dark transition test), but with no change in classic measures of anxiety [time in centre of open field, time in the light compartment of the light/dark transition test (Fujioka et al., 2014)]. One interpretation is that these subtle effects on behaviour in the *GRM3*^*−/−*^ mice reflect differences in arousal/activity levels which can influence performance on these ethological anxiety tests indirectly, rather than anxiolytic effects per se (see below for further discussion). It is also worth pointing out that Morishima et al. (2005) found a similar pattern of results for their *GRM2*^*−/−*^ mice, with increased locomotor activity observed in a number of different testing situations (including the open field test, light/dark transition test, elevated plus maze, social interaction test), but no genotype differences in performance measures more directly related to anxiety (although note that we did not replicate these effects in *GRM2*^*−/−*^ mice in the present study).

### 4.3 A role for group II metabotropic glutamate receptors at the interface between arousal and cognition

We previously demonstrated that *GRM2/3*^*−/−*^ mice show a unique fractionation within hippocampus-dependent spatial memory, leading us to suggest a role for group II mGlu receptors at the interface between arousal and cognition (Lyon et al., 2011, Marek, 2010). Our previous data suggested a possible role for these receptors in the well-established inverted U-shaped function that relates acute levels of stress and arousal with cognitive performance (Yerkes and Dodson, 1908). This was, in part, based on the dissociation that we saw between (i) impaired performance on both appetitively motivated and spontaneous tests of spatial memory, and (ii) normal performance on aversively motivated versions of the tasks (e.g. swim-escape tasks). We did not observe such a clear dissociation between these tasks in the single KO lines in the present study. For example, neither single *GRM2*^*−/−*^ nor *GRM3*^*−/−*^ mice were impaired on appetitively- or aversively-motivated spatial reference memory tasks, consistent with previous studies (Higgins et al., 2004). As previously discussed, this may reflect the opportunity for compensation and/or redundancy of function in these single KO lines.

Nevertheless, there is evidence from the *GRM2*^*−/−*^ and *GRM3*^*−/−*^ mice in the present study that is potentially consistent with our previous findings and speculations. First, it is worth noting that where cognitive deficits are apparent in the single lines in this study, they are on appetitively motivated tasks rather than aversively motivated paradigms, as is the case in the *GRM2/3*^*−/−*^ mice.

Second, the results with the *GRM3*^*−/−*^ mice on the appetitively motivated T-maze spatial working memory task revealed a biphasic effect on performance, in keeping with an inverted U-shaped function. While the *GRM3*^*−/−*^ mice were significantly impaired during the first block of 10 trials, they then performed significantly better than the WT mice during the third block of 10 trials. Two recent studies have reported short-term spatial (working) memory deficits in *GRM3*^*−/−*^ mice, although the effects were subtle and dependent, to some extent, on the precise experimental parameters being used (Fujioka et al., 2014, Lainiola et al., 2014). Indeed, Fujioka et al. (2014) found a deficit in *GRM3*^*−/−*^ mice on a T-maze, forced alternation spatial working memory task, very similar to the spatial working memory T-maze task employed here, which was present during the initial training sessions, but was then absent during later testing when delays were introduced. Thus, cognitive performance in WT and *GRM3*^*−/−*^ mice may be differentially dependent on changes in stress/arousal levels as the animal's experience with the task changes with training across time.

Third, both *GRM2*^*−/−*^ and *GRM3*^*−/−*^ mice showed alterations in the patterns of reactivity towards environmental stimuli, in line with an altered interaction between arousal state and exploration. The patterns of locomotor activity measured in photocell activity cages in both single KO lines were very different from what we have seen previously in the double KO mice, which displayed a pronounced reduction in locomotor activity during a 2 h test (Lyon et al., 2011). In the present study, *GRM3*^*−/−*^ mice displayed similar activity levels to WT controls and, if anything, were slightly *more* active in the second hour of testing. Fujioka et al. (2014) also recently reported hyperactivity in *GRM3*^*−/−*^ mice in a home cage activity monitoring system (Fujioka et al., 2014). The *GRM2*^*−/−*^ mice exhibited a biphasic effect, being hyperactive during the first 10 min bin, but exhibiting reduced activity levels as the test proceeded. This behaviour during the first 10 min of the locomotor test is potentially consistent with previous demonstrations that *GRM2*^*−/−*^ mice exhibit hyperactivity in response to novel and potentially stressful environments such as in the open field or in other ethological, unconditioned tests of anxiety (Higgins et al., 2004, Morishima et al., 2005), albeit we did not see such effects in the anxiogenic open field in our present studies. It is also notable that the *GRM2*^*−/−*^ mice displayed consistently faster running latencies (sample and choice runs) during the appetitively motivated, spatial working memory T-maze task, reminiscent of the previous result in the double KOs (Lyon et al., 2011).

Thus, although we see significant differences in activity levels between group II metabotropic glutamate receptor KO mice and their controls, the pattern and direction of these effects can vary considerably from one situation to the next. This also means comparisons across studies are difficult to make (e.g. comparing single KO data from the present study with our previous data from the double KO line). For example, although the different lines were tested in the same activity cages in the same testing room, the studies were conducted at different times (and by different researchers) making comparisons difficult. These patterns of results reflect the likely complexity of the relationship between stress/arousal levels and behavioural activity, depending on the experimental settings. We will return to this issue in the section below.

The concepts of anxiety and arousal, although far from identical, are often closely coupled, although the interaction between them is likely to be complex. Although we have not seen clear effects on anxiety in any of the KO mice, it is known that mGlu2/3 agonists – and, in some studies, antagonists – produce robust anxiolytic effects which are dependent on these receptors (Linden et al., 2005). Changes in stress/arousal levels have profound effects on anxiety, but increases in arousal are also an important component of the anxiety response. Neuropsychological accounts have suggested that anxiety is a response to situations of conflict or uncertainty, and that when such situations are detected then a constellation of behavioural responses are evoked, including increases in attention and arousal processes (Gray, 1982, Gray and McNaughton, 2000). Thus, a more general account in which group II metabotropic glutamate receptors play an important role in mediating the effects of changes in arousal on behaviour, could easily accommodate the role of these receptors in anxiety.

### 4.4 Studying single and double knockout lines

The behavioural consequences of deleting either mGlu2 or mGlu3 alone are less than those associated with deleting both receptors, consistent with the idea that there is redundancy of function and/or compensation in the single KO lines (Lyon et al., 2008). Although it is difficult to make comparisons across studies run at different times, and by different experimenters, the phenotypes of the single KOs were less pronounced (if present at all), across a number of different behavioural tasks. It is also worth pointing out that the apparatus, testing rooms and test running orders were comparable for both the single and double KO lines. Thus, this pattern of results generalised across a variety of different test settings.

An important caveat relates to the breeding strategy for the double KO mice. Generating sufficient numbers of double KO mice necessitated, for both financial and ethical reasons, the use of separate lines for double KOs and controls. Thus, unlike the case for the *GRM2*^*−/−*^ and *GRM3*^*−/−*^ lines, the *GRM2/3*^*−/−*^ controls are non-littermate WTs, although they were bred in the same holding room, in the same facility, at the same time. This is a common practice with double KO mouse studies, but as a result we cannot rule out the possibility that the *GRM2/3*^*−/−*^ mice results are affected by differences in genetic background, epigenetic effects or parental behaviour (for discussion see Lyon et al., 2011). In the context of the present study, however, this may be less of an issue, as the major novel finding with these mice was a negative one: the lack of an anxiety phenotype. Therefore, any confounding effect resulting from the breeding strategy would have to be both opposite in direction and of equal magnitude in order to cancel out any putative effect of the double KO.

A further, additional caveat relates to the use of constitutive, permanent KOs in which the gene (or genes) of interest are absent both throughout development and in adulthood. With these mice it is impossible to separate effects arising from the absence of the gene on developmental processes, from effects arising due to the absence of the gene at the time of testing. Therefore, we cannot exclude the possibility that when we do see effects in our group II metabotropic glutamate receptor KO animals, these phenotypes could reflect a developmental disruption. We also cannot exclude the possibility that the absence of a gene (or genes) throughout the entire lifetime of the animal could result in effective compensatory changes which might produce a null phenotype (e.g. for anxiety), despite a role for these receptors in normal animals. Inducible KO mice provide a possible future approach for resolving these issues.

### 4.5 Studying the relationship between arousal and cognition

We have argued here and elsewhere (Lyon et al., 2011; see also Marek, 2010), that group II metabotropic glutamate receptors may play an important role at the interface between arousal and behavioural performance, according to an inverted U-shaped function (Yerkes and Dodson, 1908). This is potentially consistent with the biphasic effects on activity levels that are seen in the KO mice in different experimental settings throughout the present study (see also Lyon et al., 2011). However, these studies illustrate the difficulties of studying experimental manipulations that affect the clearly non-linear interaction between arousal processes and behavioural performance. Depending on the baseline levels of stress and/or arousal generated by the experimental situation, it may be possible to generate any one of three results (group A > group B, group A = group B, group A < group B). Therefore, although the inverted U-shaped function is an attractive post-hoc explanation for much of the data generated here and elsewhere (Lyon et al., 2011), it is very difficult in practice to predict *a priori* the direction and/or magnitude of an effect in any given experiment or, therefore, come up with a falsifiable hypothesis. Arousal/stress levels and hence performance could be affected by a whole host of factors (e.g. age, gender, housing conditions, experimenter, time of day, prior history, experimental test conditions, behaviour of other animals, presence/absence of injection, level of food restriction), and this could be especially pertinent for hand run tests (*e.g*. ethological anxiety tests, maze tests of learning and memory) compared with operant tasks. Ultimately, independent physiological measures of stress/arousal levels (e.g. corticosterone levels, indicators of sympathetic tone such as heart rate or blood pressure), taken during the behavioural test itself, may be necessary to understand fully the complex relationship between stress, arousal and cognitive performance, and the role which group II mGlus play in these processes.

### 4.6 Conclusions

In summary, the behavioural phenotypes of the *GRM2*^*−/−*^ and *GRM3*^*−/−*^ mice were less pronounced than those of the double *GRM2/3*^*−/−*^ mice, consistent with the possibility of redundancy of function and/or compensation in the single KO lines. However, there was no clear effect on measures of anxiety in either the single or double KO mice. Nevertheless, behavioural phenotypes were observed in these animals which are potentially consistent with a role for group II metabotropic glutamate receptors at the interface between arousal processes and behavioural performance.
